# Effectiveness of lasers and aPDT in elimination of intraoral halitosis: a systematic review based on clinical trials

**DOI:** 10.1007/s10103-022-03656-3

**Published:** 2022-11-24

**Authors:** Agnieszka Woźniak, Jacek Matys, Kinga Grzech-Leśniak

**Affiliations:** 1 Wroclaw Medical University, Wroclaw, Poland; 2 Laser Laboratory Oral Surgery Department, Wroclaw Medical University, Wroclaw, Poland; 3 Department of Orthodontics, Technische Universitat Dresden, 01307 Dresden, Germany; 4 Department of Periodontics School of Dentistry, Virginia Commonwealth University, VCU, Richmond, VA USA

**Keywords:** Bad breath, Fetor ex ore, Halitosis, Laser, Oral malodor

## Abstract

In recent years, there has been increasing interest in research showing positive results in antimicrobial photodynamic therapy (aPDT) and laser therapy (LT) in dentistry. The authors of this review tried to answer the question: “Is the effectiveness of lasers and aPDT in the elimination of intraoral halitosis possible?” For this purpose, the electronic database of PubMed and Cochrane Library were searched until September 2021 using a combination of different keywords: (bad breath OR fetor ex ore OR halitosis OR oral malodor) AND (laser OR PDT OR PACT OR photodynamic inactivation OR photodynamic therapy OR photodynamic antimicrobial chemotherapy). Initially, 83 studies were identified. A total of 9 articles were qualified after the application of the eligibility criteria. Eight works concerned aPDT treatment, and only one dedicated to the Er,Cr:YSGG laser. A significant reduction in halitosis occurred immediately after both LT and aPDT. The review found the confirmation of the effectiveness of laser therapy in reducing the number of volatile sulfur compounds (VSC) and the amount of anaerobic bacteria responsible for VSC formation. In most studies, a positive effect was observed for a 1-week follow-up. Laser therapy (aPDT, Er,Cr:YSGG) effectively eliminates microorganisms that produce volatile compounds and can effectively eliminate bad breath for the longer period of time than traditional methods of combatting this ailment.

## Introduction

Halitosis describes any unpleasant odor of exhaled air, regardless of its source. Other commonly used names are oral malodor, bad breath, and fetor ex ore. The incidence of this disease in the population amounts to 31.8%, with 85–90% of cases having its origins in the oral cavity [1, 2]. Halitosis affects the quality of social life, leading to embarrassment and psychological withdrawal. It is caused by the accumulation of decomposed food debris on the back of the tongue, in its pits and between the filamentous papillae. It is also affected by salivary proteins, exfoliating epithelium that is broken down by bacteria in the mouth. The classification of intraoral halitosis (IOH) consists of three basic groups: true, pseudohalitosis, and halitophobia [3]. True halitosis has been divided into the physiological one, in which there is no disease process, and the pathological one, which occurs in inflammation of the gums, periodontium, tonsils, xerostomia, and tooth decay. Pseudohalitosis is a condition in which patients experience an unpleasant odor, but no one else confirms it. Halitophobia is a consequence of the treatment of halitosis and is associated with the fear of recurrence of unpleasant symptoms [3].

The main components of bad breath are volatile sulfur compounds (VSC), i.e., hydrogen sulfide, methyl mercaptan, dimethyl sulfide, and other volatile organic compounds resulting from metabolic changes. VSC in the oral cavity are produced mainly by anaerobic bacteria, which are found, among others, on the dorsum of the uvula (51%), in the gingival pockets, in the interdental plate, and tonsils [4]. Bacteria, including *Solobacterium morei*, are responsible for biofilm formation on the tissues in the oral cavity and the breakdown of amino acids, mainly methionine and cysteine, from which VSC are secreted [4]. Other bacteria involved in halitosis include *Treponema denticola*, *Porphyromonas gingivalis*, *Fusobacterium nucleatum*, *Capnocytophaga gingivalis*, *Prevotella intermedia*, and *Peptostreptococcus micros* [4]. Measurements of VSC quantity in ppb (part per billion) are carried out with devices such as Halimeter, Breathtron, and oral OralChroma [5, 6]. Until the outbreak of the COVID-19 pandemic, the subjective organoleptic method introduced by Rosenberg and McCulloch [7], involving the patient blowing air from the mouth into a tube, and the air odor being judged by the use of the sense of smell of the evaluator (clinician) was the gold standard for assessing halitosis.

There are no standard methods of treating unpleasant odor from the mouth, as it can be a symptom associated with many general diseases (extraoral halitosis, EOH); however, approximately 10–15% can be related to a mouth disease only (IOH) [1]. Patients with halitosis use various odor-neutralizing agents. These are mouthwashes, chewing gums, lozenges, toothpaste containing mostly alcohol, chlorhexidine, zinc, and essential oils. The use of a tongue scraper is also recommended. However, these methods work efficiently for a short period of time only. Other safe methods of removing or decreasing the malodor are being sought. The high-power lasers, i.e., Er:YAG, Er,Cr:YSGG, and Nd:YAG, which are applicable for disinfection and cleansing tissues by evaporating the water they contain can be one of them. Also, antimicrobial photodynamic therapy (aPDT), which consists of the activation of the photosensitizer by an appropriate light wavelength, eliminates pathogens by using singlet oxygen or other reactive forms of oxygen [8–13]. Thanks to various laser systems, researchers and clinicians can reduce the number of microorganisms in the oral cavity [14].

This study aimed to provide a comprehensive literature review and evaluate the effectiveness of various laser wavelengths and aPDT in the treatment of halitosis in vivo in a human model.

## Materials and methods

### Focused question

The question posed by the authors of this review is as follows: “Is it possible to treat halitosis effectively with lasers and aPDT in healthy patients?”.

### Protocol

**Fig. 1:**
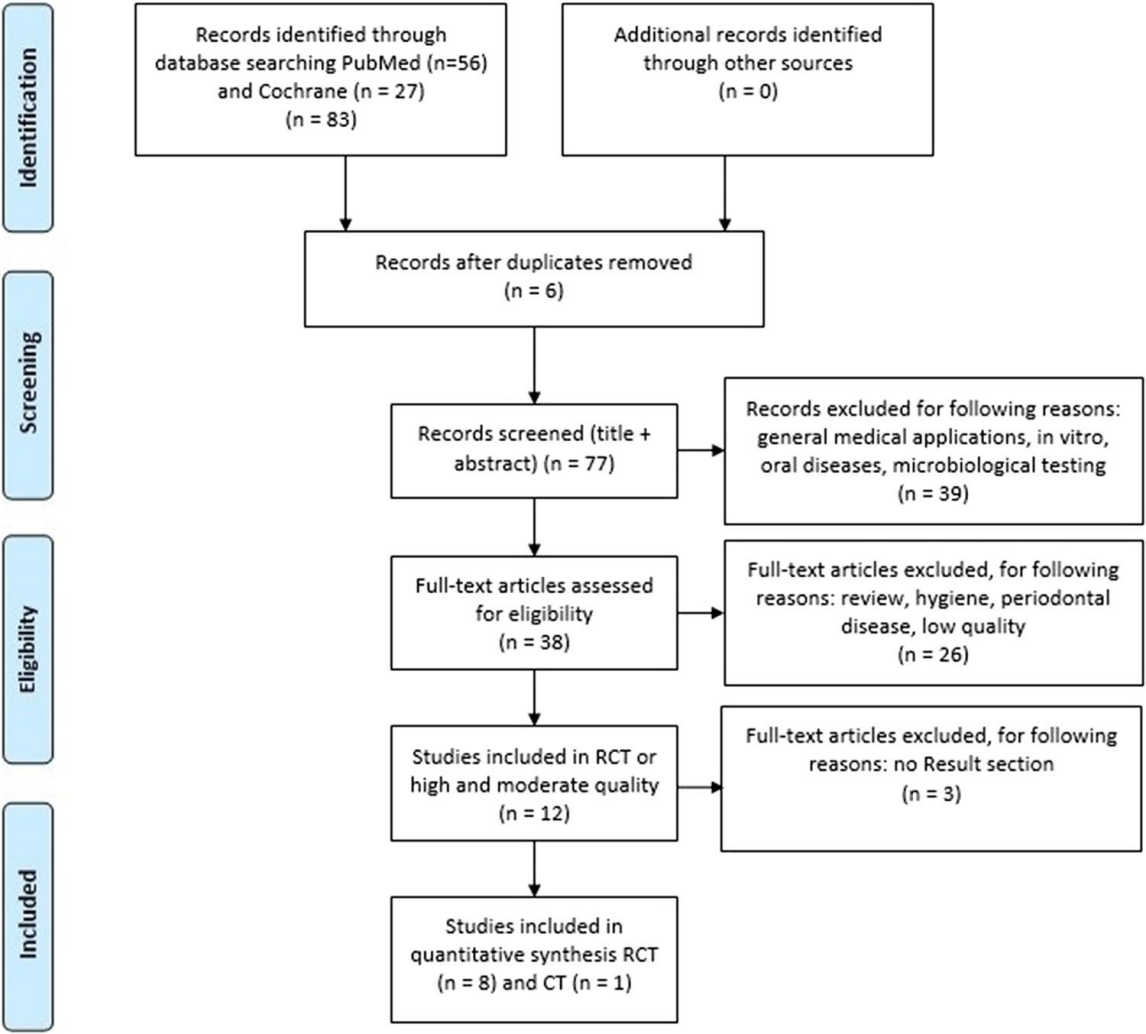
PRISMA flowchart presenting the criteria for the included studies

According to the PRISMA scheme, the details of the selection criteria are presented in Fig. 1

### Eligibility criteria

The articles’ selection criteria for the review included studies of healthy subjects over 12 years of age participating in randomized clinical trials (RCT) or clinical trials (CT) with a minimum observation period of more than 7 days.

Non-English language articles, reviews, and opinions that did not consider laser treatment of bad breath were excluded. Articles related to the treatment of patients with systemic diseases accompanied by halitosis and to the cases of advanced periodontal disease were also excluded.

Inclusion criteria:

- Use of a lasers or aPDT
- Number of patients not less than 10 per group
- Randomized clinical trial
- Clinical trial
- Laser treatment of halitosis in generally healthy subjects
- The effect of the laser on the bacterial flora in the oral cavity
- A minimum of one week of observation after laser/aPDT use
- The use of various photosensitizers in aPDT
- In vivo studies

Exclusion criteria:

- Non-randomized studies
- Examinations of patients after laser treatment with general diseases, except for MS (multiple sclerosis)
- Number of patients less than 10 per group
- Patients age less than 12 years
- Laser treatment of advanced periodontal diseases and other oral diseases
- Change of the bacterial flora in the oral cavity without the use of a laser
- Duplicated articles
- No use of a laser
- In vitro studies

### Search strategy

An electronic screening of PubMed and the Cochrane Central Register of Controlled Trials (CENTRAL) databases from 1994 to September 2021 was done. A following combination of keywords was used: (halitosis OR fetor ex ore OR bad breath OR oral malodor) AND (laser OR aPDT or PACT OR photodynamic inactivation OR photodynamic therapy OR photodynamic antimicrobial chemotherapy). The search strategy was limited to studies that met the eligibility criteria. Articles with fully available texts were taken into account.

### Information sources, search strategy, and study selection

Two reviewers (A. W., J. M.) independently extracted data from articles that met the inclusion criteria, and the third one (K. G. L.) checked the accuracy of the selection and resolved the disputed decisions. The following data was used: first author, year of publication, title, study design, laser type, laser parameters, photosensitizer type and concentration, incubation time, study groups, study results, and changes in the amount of VSC in exhaled air before and after treatment. The extracted data was saved in a standard Excel sheet.

### Quality assessment

Two blinded reviewers filtered the studies individually and independently to assess the quality of each included study. The study analysis and implementation were based on the following criteria: description of laser parameters and laser type, the use of laser power meter to standardize lasers parameters, detailed treatment protocol, randomization, blinding, and control group, at least 1-month follow-up. The scoring range was from 0 to 9 points. The higher the result, the higher the quality of the test. Any disagreements were resolved through discussion until reaching consensus.

### Risk of bias

The scores of each study were calculated, and overall the estimate risk of bias (low, moderate, high) was made for each included study, as recommended in the Cochrane Handbook for Systematic Reviews of Interventions [15]. The risk of error based on sums of answers: yes—1 or no—0 was determined.

The total number of 1-yes answers shows us the degree of systematic error, assessed with scoring limits: 0–3 high risk, 4–6 moderate risk, and 7–9 low risk. The higher the result, the higher the quality of the test.

## Results

### Study selection

The main aim of this review was to evaluate the effectiveness of various laser wavelengths and aPDT in the treatment of halitosis in vivo in a human model.

Fifty-six articles had been found in the PubMed search engine and 27 in the Cochrane Library one. Six repeating research papers from both search engines were excluded. After using filters for randomized trials (RCT) and clinical trials (CT), the number of articles were reduced to 9. The review of articles included 8 randomized clinical trials [16–23] and 1 clinical trial [24]. In the treatment of halitosis, 8 studies concerned the use of diode lasers with a photosensitizer [16–21, 23, 24], while 1 of them involved the Er,Cr:YSGG laser [22].

The best results in reducing VSCs were shown in papers evaluating tongue scraper and aPDT [16, 19, 20, 23]. Most studies used methylene blue as a photosensitizer [16–21, 24], activated with a 660 nm wavelength. One article described the use of bixa orellana (other names urucum, arnota proper) as a yellow photosensitizer activated with a laser with a wavelength of 395–480 nm [23].

The influence of Er,Cr:YSGG on the level of VSC in patients without periodontal disease was described in one study [22] (Table 1).

**Table 1:** Characteristics of lasers used for treatments

N^o^	Laser type	Wavelength (nm)	Power (W)	Laser therapy	Reference number
1	Diode laser	660	0.1	aPDT	Costa da Mota et al. [16]
2	Diode laser	395–480	0.48	aPDT	Goncalves et al. [23]
3	Diode laser	660	0.1	aPDT	Llanos do Vale et al. [17]
4	Diode laser	660	0.1	aPDT	Romero et al. [18]
5	Diode laser	660	0.4	aPDT	Ciarcia et al. [19]
6	Diode laser	660	0.1	aPDT	Goncalves et al. [24]
7	Diode laser	660	0.1	aPDT	Lopes et al. [20]
8	Diode laser	660	0.1	aPDT	Labban et al. [21]
9	Er,Cr:YSGG laser	2780	4	Vaporization/debridement	Krespi et al. [22]

### Results of individual studies

The study by Romero et al. [18] with the longest follow-up showed a reduction in halitosis after applying aPDT and a tongue scraper, and the effect was maintained throughout the 3-month observation period. Most studies reported an effect of reducing malodor immediately after treatment, and it lasted for a week [16–18, 20, 21, 23, 24]. Labban et al. [21] described the reduction in *Porphyromonas gingivalis* and VSC over 1 month of follow-up.

Krespi et al. [22] in his research on the Er,Cr:YSGG laser described a reduction in VSC in exhaled air, the amount of anaerobic and aerobic bacteria in a microbiological study, and an improvement in the appearance of the tongue. The positive results were maintained until the end of the 1-month observation period (Table 2).

**Table 2:** Characteristics and results of included studies

Authors	Study type/number of patients/level of VSC	Groups	Laser (nm) + photosensitizer	Protocol aPDT	Study observation—time	Results
1. Costa da Mota et al. [16]	RCT 46 patients	G1-aPDT (*n* = 15), G2- tongue scraper (*n* = 15) G3 (*n* = 16)	660 nm + methylene blue MB (0.05 mg/ml)	*P* = 100 mW, E = 9 J, *t* = 90 s per point, density *P* = 3.5 W/cm^2^, density *E* = 320 J/cm^2^, 6 points, applicator 0.028 cm^2^, BM- incubation 5 min, irradiation distance: 1 cm from tongue	1 session, 1 week, the treatment microbiological analysis before and immediately after	G3—highest reduction in VSC, G2- lowest reduction in VSC After 1 week, there were no significant differences in quantity of VSC in particular groups. No significant differences in the microbiological analysis
2. Goncalves et al. [23]	RCT 44 patients	G1 = 15, G2 = 14, G3 = 15 G1 = aPDT + BO (bixa orellana) G2 = tongue scraper G3 = aPDT + scraper	395–480 nm BO (bixa orellana), 20% solution	*P* = 480 mW, E = 9.6 J, *t* = 20 s/per point, density *P* = 153 mW/cm^2^, density *E* = 6.37 J/cm^2^, applicator 3.14 cm^2^, irradiation points = 6, contact method, incubation on the tongue for 2 min	1 session, immediately after the treatment, 1 week	Quantity of VSC diminished in all groups. There were no significant differences in G1 and G3 groups. One week after the treatment, no significant differences were noticed with respect to the level of VSC
3. Llanos do Vale et al. [17]	RCT 45 patients	G1 = 22 G2 = 23 G1- tongue scraper G2- aPDT	660 nm, methylene blue—MB (0.005%)	*P* = 100 mW, *E* = 9 J, *t* = 90 s, density *P* = 35,368 mW/cm^2^, density *E* = 3183 J/cm^2^, 6 points irradiated with a contact method, applicator = 0.00287 cm^2^, MB—5 min on the tongue and rinsed with saline	1 session 1 week (VSC level)	aPDT significantly lowered levels of VSC
4. Romero et al. [18]	RCT 40 patients Single-blind trial	G1 = 20 G2 = 20 G1- aPDT G2- tongue scraper	660 nm methylene blue 0,005%	*P* = 100 mW *E* = 9 J per point *t* = 1 min density *P* = 3537 mW/cm^2^ density *E* = 318 J/cm^2^ 6 points	1 session, results immediately after the treatment 1 week 90 days (VSC measurement)	The VSC values were 2 (scraper) or 3 (aPDT) times lower than the baseline value after 1 week and after 3 months. The most significant improvement immediately after the treatment was observed
5. Ciarcia et al. [19]	RCT 39 patients	G1 = 13 G2 = 13 G3 = 13 G1-aPDT G2- tongue scraper G3- aPDT i scraper	660 nm methylene blue 0,005%	*P* = 400 mW *E* = 36 J *t* = 90 s *P* = 1039.4 mW/cm^2^ density *E* = 93.5 J/cm^2^ 4 irradiated points, distance: 2 cm, for 2 min	1 session, immediately after the treatment 1 week 14 days 30 days (VSC level)	Directly after the treatment the significant reduction in VSC was observed No data available after 7, 14, and 30 days
6. Goncalves et al. [24]	CT 60 patients	G1 = 20 G2 = 20 G1-with MS G2—healthy	660 nm methylene blue 0.005%	*P* = 100 mW *E* = 9 J *t* = 90 s for a point density *P* = 3537 mW/cm^2^ density E = 320 J/cm^2^ applicator- 0.094 cm^2^ 6 points irradiated with contact method MB on the tongue for 5 min	1 session examination immediately after the treatment	Level of VSC diminished in both groups
7. Lopes et al. [20]	RCT 45 patients	G1 = 16 G2 = 15 G3 = 14 G1-aPDT G2- tongue scraper G3- aPDT + scraper	660 nm methylene blue 0.005%	*P* = 100 mW *E* = 9 J *T* = 90 s W/cm^2^ density *E* = 317.43 J/cm^2^ Applicator -0.02835 cm^2^ 6 points separated by 1 cm were irradiated with the contact method, *P* = 3537 mW/cm^2^ Methylene blue 5 min. on the tongue	1 session 1 h and 24 h after treatment (VSC level)	There was a significant reduction in VSC in all groups, the highest result of the reduction of VSC was shown in group G3
8. Labban et al. [21]	RCT 40 patients	G1 = 20 G2 = 20 G1- tongue scraper G2-scraper + aPDT	Laser 660 nm, methylene blue 0.005%	*P* = 100 mW, density *P* = 3527 mW/cm2, *E* = 9 J 6 points on the tongue and on the prosthesis, distance – 1 cm, MB – 5 min	1 session, 5 days after, 15 days after, 30 days after treatment, the level of VSC was measured, microbiological test for the presence of Porphyromonas gingivalis was performed, OHIP-14 profile	Decrease in the level of H2S, which was sustained during the month of observation, decrease in the amount of *P. gingivalis* (highest after 5 days)
9. Krespi et al. [22]	RCT 60 patients	G1 = 30 G2 = 30 G1- tongue scraper G2 laser	2780 nm Er,Cr:YSGG	*P* = 4 W *E* = 100 mJ *f* = 40 Hz *t* = 60 s Pulse width—60 µs Air -10% Water—5% Tip—MC12 distance from the tongue 3 mm Density *E* = 3 J/cm^2^ 10 passes	1 session/1 month The level of VSC was measured; a microbiological test has been performed, the appearance of the tongue was assessed	Decrease in VSC and the number of anaerobic and aerobic bacteria, improvement in the appearance of the tongue, sustained throughout the observation period

*RCT*, randomized clinical trial; *aPDT*, antimicrobial photodynamic therapy; *P*, power; *E*, energy; *MS*, multiple sclerosis

### Quality assessment

Seven articles showed a low 7–9 bias [16, 18–23]. Two studies were in the moderate range of 4–6 of bias [17, 24]. None of the articles was classified as of a high risk of error (Table 3).

**Table 3:** Quality assessment of the included studies

Criteria	Authors								
	Costa da Mota [16]	Goncalves [23]	Llanos do Vale [17]	Romero [18]	Ciarcia [19]	Goncalves [24]	Lopes [22]	Labban [21]	Krespi [22]
Randomization	1	1	1	1	1	0	1	1	1
Blinding	1	0	0	0	0	0	0	1	0
Sample size calculation	1	1	1	1	1	1	1	1	1
Control group	1	1	1	1	1	1	1	1	1
Laser parameters: (power, energy, density) applicator type	1	1	1	1	1	1	1	1	1
Power meter use	1	1	0	1	1	0	1	0	1
Laser type (wavelength)	1	1	1	1	1	1	1	1	1
Detailed treatment protocol	1	1	1	1	1	1	1	1	1
At least 1-month follow-up	0	0	0	1	0	0	0	1	1
Total score	8	7	6	8	7	5	7	8	8
Risk of bias	low	low	medium	low	low	medium	low	low	low

## Discussion

All of the included studies reported the results of the reduction of bad breath coming from the analysis of volatile sulfur components detection devices [16–24]. The most commonly used method of VSC measurement was gas chromatography (Oral Chroma TM Abilit, Japan) [16–21, 23, 24] and halimetric analysis of hydrogen sulfide (Halimeter, Interscan Corporation, Chatsworth, USA) [22]. Additionally, Krespi et al. [22] determined breath quality by analyzing patient-perceived results using a visual analog scale (VAS) and the tongue’s appearance before and after treatment. The results of included studies were reported immediately after treatment [16–24], and the maximum follow-up was 3 months [18]. Most included studies showed a benefit of using aPDT in generally healthy patients with halitosis [16–21, 23, 24]. Only in one study by Krespi et al. [22] the application of high-level laser (Er,Cr:YSGG) reduced the amount of VSC, the number of anaerobic and aerobic bacteria, and improved the tongue’s appearance.

Almost all of the included studies indicated aPDT as an efficient method in treating malodor [16–21, 23, 24]. Antibacterial photodynamic therapy is a process in which non-toxic photosensitizing substances and oxygen are combined with the appropriate wavelength of light [9, 11]. That phenomenon leads to the formation of reactive oxygen species that are deadly against bacteria, viruses, and fungi [11, 25]. PDT appears to represent an efficacious alternative modality for treating localized microbial infections through the in situ application of the photosensitizer followed by irradiation of the photosensitizer-loaded infected area [14]. In most studies on the effectiveness of aPDT, methylene blue (MB) and a laser with a wavelength of 660 nm were used [16–21, 23, 24]. One study focused on the use of bixa orellana with a laser wavelength of 395–480 nm [22]. All laser wavelengths combined with both photosensitizers (methylene blue and bixa orellana) used in included studies allowed for a significant reduction of the VCS amount.

Many studies confirmed that halitosis is caused by volatile sulfur components, which are the product of metabolic changes in anaerobic bacteria [4, 26–28]. A significant number of these bacteria are located on the back of the tongue. Mechanical cleaning of the tongue with a scraper reduces the amount of bacterial wastes, but does not significantly reduce the amount of microbes [29]. In their study, Mantilla et al. [29] did not observe a relationship between the appearance of the tongue and salivary bacterial load. The elimination of the number of bacteria and the change in their quality have had an impact on the reduction of unpleasant breath (malodor) [30]. In their study, Labban et al. [21] proved a significant decrease in the amount of *Porphyromonas gingivalis* (highest after five days). Moreover, Krespi et al. [22] observed a decrease in the total number of bacteria when using Er,Cr:YSGG laser. The laser treatment was significantly more effective immediately after the treatment than the tongue scarper at diminishing both anaerobic and aerobic cultures [22].

Last but not least, only one article assessed the influence of the Er,Cr:YSGG high-power laser on the level of VSC in the oral cavity. Krespi et al. [22] reported positive outcomes for all tested variables after laser treatment. The authors pointed out that the sustained reduction of VSC concentration due to laser treatment compared to that of the tongue scraping was significant. The results were maintained for one month of follow-up. The microbiological analysis of the tongue, its appearance, and the patient’s subjective feelings were assessed by using the HALT test. The decrease in total anaerobic bacteria from baseline to 1 month remained significantly higher for the laser treatment group than for the control group. The Er,Cr:YSGG laser effectively removed biofilm through light and water dual action [22]. This physical property damages water-rich cells, which is essential in eradicating pathogens [10]. Further studies should be conducted to examine whether laser-assisted halitosis treatment combined with various lasers (Er:YAG, Nd:YAG) improves the VCS and total microbial count.

## Conclusions

Laser therapy (aPDT, Er,Cr:YSGG) effectively eliminates microorganisms that produce volatile compounds and it can effectively eliminate bad breath for the longer period of time than traditional methods of curing this ailment. Halitosis is an underestimated problem of the global population. It has a significant impact on quality of life and social withdrawal. There is a need to resolve this social problem, looking for minimally invasive treatments.
